# Artifacts and Anatomic Variations in Optical Coherence Tomography

**DOI:** 10.4274/tjo.galenos.2019.78000

**Published:** 2020-04-29

**Authors:** Atilla Bayer, Ahmet Akman

**Affiliations:** 1 Dünyagöz Hospital, Ankara, Turkey; 2 Private Practice, Ankara, Turkey

**Keywords:** Optical coherence tomography, OCT artifacts, OCT anatomic variations, red disease, green disease

## Abstract

In recent years, ophthalmologists widely depend on optical coherence tomography (OCT), which is an objective, reliable, and repeatable structural test for both early diagnosis of glaucoma and detecting progression of the disease. Using this technology, it is now possible to take measures of various anatomic structures and layers of the optic nerve head, peripapillary retinal nerve fiber layer, and macular area. Although OCT has these powerful capabilities in general, anatomical variations, artifacts related to the ocular pathologies, and issues with image acquisition can be present in up to one-third of scans. These anatomical variations and artifacts can be misleading to an interpreter and may lead to erroneous conclusions. This review focuses on the realization and prevention of most common anatomical variations and artifacts observed with OCT imaging. The concepts of floor effect and red and green diseases are also investigated.

## Introduction

Optical coherence tomography (OCT) has been widely used in recent years for both the diagnosis and follow-up of glaucoma, as well as in other areas of ophthalmology.^1,2,3,4^ When initiating treatment for patients with glaucoma, suspected glaucoma, or ocular hypertension, ophthalmologists generally base their decisions on OCT results. As with any newly introduced diagnostic method, it may take time to understand the limitations and sources of error of OCT. Evaluating results with knowledge of these limits and sources of errors will make OCT results more reliable in the diagnosis of new cases and analysis of progression.

For a sound evaluation of OCT data, physicians should not limit themselves to the colored images, tables, or maps that compare patient data with the normative database. The classifications in these images, tables, and maps are based on the manufacturer’s normative database and are open to various sources of error. For this reason, when evaluating OCT reports the physician should examine en-face images, temporal-superior-nasal-inferior-temporal (TSNIT) profiles, and the patient’s unprocessed scan results to ensure accurate data analysis. Knowing potential sources of error is critical at this stage to be able to make a correct decision.^5,6,7,8^ This is the only way we can distinguish anatomical variations and artifacts from true glaucomatous damage. Because retinal nerve fiber layer (RNFL) thickness can vary by race, this must also be considered starting from patient registration.^9^

Overlooking OCT artifacts can cause patients who actually have glaucomatous damage to be evaluated as normal (i.e., false-negative diagnosis) or conversely, lead to false-positive diagnosis of individuals without glaucoma. False-positive diagnoses can lead to years of unnecessary treatment and follow-up. In addition to the adverse effects and expense associated with treatment, being diagnosed with a potentially blinding condition can cause patients serious psychological distress.^10^

The aim of this review is to examine common OCT artifacts and anatomical variations that can lead to misdiagnosis and explain how we can correct these errors in cases where they may occur. The most widely used OCT images are those of the Cirrus HD-OCT (Carl Zeiss Meditec, Dublin, CA, USA) and Spectralis OCT (Heidelberg Engineering Inc., Heidelberg, Germany) instruments.

## Causes and Mechanisms of OCT Artifacts

### Floor Effect

Compared to early and moderate glaucoma, OCT is less useful in advanced cases. In advanced glaucoma, only the retinal vascular structures and glial cells remain due to RNFL loss.^5,6^ In other words, glaucoma progression analysis with OCT is no longer possible at this stage, and visual field should be the focus during follow-up.

In the OCT devices currently in use, RNFL thicknesses less than 30 µm cannot be obtained, even in a small area.^5^ If a lower RNFL thickness measurement is observed, the physician should carefully examine the scan for artifacts.

### Red and Green Disease

Red and green are the colors used by OCT manufacturers to indicate whether various parameters of a patient are within normal limits compared to a normative database. Red disease refers to a false-positive disease diagnosis due to the device incorrectly indicating abnormality (red) in the corresponding image when there is no damage.^5,11^ In contrast, green disease is when the software interprets actual glaucomatous damage as normal (green), leading to a false-negative diagnosis.^12^

In addition to device-related artifacts, it should also be borne in mind that red and green color assignments in OCT reports stem from the normative database used. As the normative databases used by manufacturers do not account for variations related to high refractive error, the pediatric age group, and race, results may be erroneous in some patient groups.

## Common OCT Artifacts and Anatomical Variations

### Imaging ArtifactsImaging Artifacts

### Poor Image Quality

**Figure 1:**
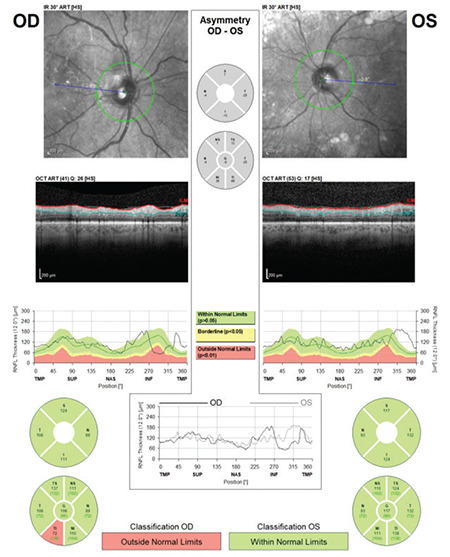
(Spectralis OCT) Example of a scan with low quality score for the left eye of a patient with cataract. Note that the quality coefficient (Q) is 26 on the right and 17 on the left. In the RNFL profile image of the left eye, RNFL thickness measurements are artificially high in the inferior and temporal regions due to incorrect detection of the RNFL border by the device algorithm RNFL: Retinal nerve fiber layer, OCT: Optical coherence tomography

A high-quality scan is essential for a reliable OCT result. Each OCT system has its own system for assessing image quality. For example, Cirrus HD-OCT uses the “signal strength” parameter for this purpose and repeating the scan is recommended if signal strength is below 6. Spectralis OCT uses a quality score, or the “Q” coefficient, for the same purpose; values less than 20 require repetition of the test. Signal strength may be low in patients with dry eye, refractive media opacities such as nephelion or cataract, and fixation disorders (Figure 1). Low signal strength may also occur if the lens of the OCT device has not been cleaned, the device is heavily used, or the technician is inexperienced. In heavily used devices, dirty lenses and reduced laser emission power may result in poor image quality. Poor scanning quality can lead to inaccurate RNFL thickness measurements.^13,14,15^

**Figure 2:**
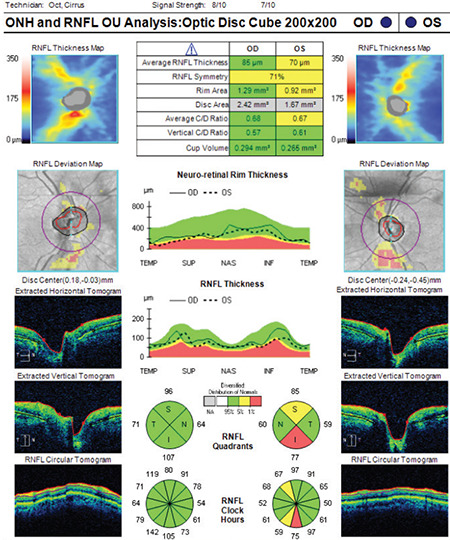
(Cirrus HD-OCT) Although signal strength is within normal range (≥ 6), there are significant motion artifacts in the deviation map of the right eye (note the breaks in the blood vessels). Similar motion artifacts are also present in the RNFL deviation map of the left eye. Average RNFL thickness is 85 μm in the right eye and 70 μm in the left eye. While the TSNIT profile and RNFL classification are within normal limits in the right eye, there are abnormalities in some sectors of the left eye RNFL: Retinal nerve fiber layer, OCT: Optical coherence tomography

However, a quality control parameter within acceptable limits should not be interpreted as everything being in order. Other possible artifacts must also be ruled out. One of these is motion artifacts. Motion artifacts occur when patients move their eyes during the scan. These artifacts manifest as breaks in deviation maps or infrared reflectance (IR) images (Figure 2). It may be noticed during scanning that the disc or macula is not well centered. In such cases, the patient should be informed and the test repeated, using an external fixation point if necessary.

### Segmentation Errors

**Figure 3:**
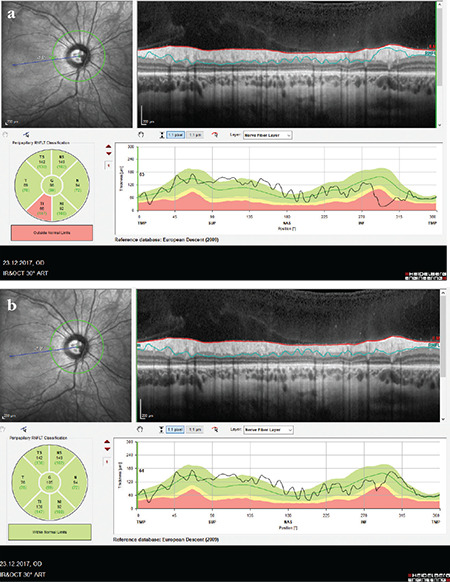
(Spectralis OCT) Segmentation artifact in the inferotemporal region in a myopic patient. Due to incorrect detection of the RNFL border by the device (in the upper right RNFL profile image), RNFL thickness measurement was artificially low in an area of approximately one sector, and peripapillary RNFL thickness was classified as abnormal in that sector. When the RNFL border is manually shifted to its normal position using the device settings, RNFL thickness returns to normal values and peripapillary RNFL thickness in the inferotemporal sector is also classified as normal RNFL: Retinal nerve fiber layer, OCT: Optical coherence tomography

All OCT systems have segmentation or layer-seeking algorithms to enable analysis of a target retinal layer. Segmentation errors occur when the software is unable to correctly distinguish the retinal layers. In such cases, the RNFL or other retinal layer being assessed is measured as thicker or, more commonly, thinner than it actually is. On RNFL thickness maps and graphs, large areas are marked red or green, and sometimes white (Figure 3a). When the RNFL border is manually moved to its normal position using the device settings, global and sectoral RNFL thickness values return to normal (Figure 3b). The floor effect should also be kept in mind for measurements of very low (thinner than 30 µm) RNFL thicknesses.

**Figure 4:**
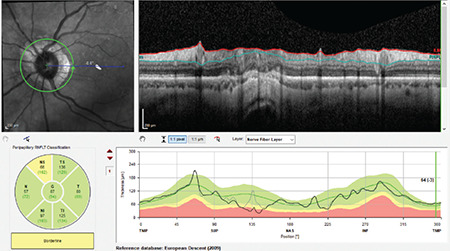
(Spectralis OCT) Segmentation error resulting from the passage of the scanning ring over an atrophic area in the superonasal region of a myopic patient with peripapillary atrophy. Values on the TSNIT profile are close to zero and peripapillary RNFL classification is borderline in that area RNFL: Retinal nerve fiber layer, OCT: Optical coherence tomography

In patients with high myopia and tilted discs, symmetric and bilateral nasal RNFL thinning (red disease) may be observed. This is due to temporal displacement of the RNFL bundles and vascular structures in myopic patients. The magnification effect caused by longer than normal axial length may also contribute to this measurement error. Peripapillary atrophy, which frequently accompanies myopia, is another cause of segmentation errors. Upon careful examination of the regions covered by the peripapillary scanning ring, which is normally 3.46 mm in diameter, it can be seen that the section passes through an area of peripapillary atrophy (Figure 4). In this case, the scan should be repeated using a larger ring (4.1 mm or 4.7 mm, as in Spectralis OCT) or the data from macular and optic nerve head analyses should be used.

Myelinated nerve fibers are another common anomaly. Thickly myelinated nerve fibers can hide glaucomatous RNFL loss and may cause inaccurate segmentation and green disease. Again, in such cases the peripapillary scan should be repeated with larger ring diameter, or the macular and optic nerve head analyses should be taken into account.

### Patient-based Artifacts

Patient-based artifacts are the most challenging for physicians. It is common to see red areas in the results of a reliable OCT scan performed on a young and healthy person with no known ocular disease. Some of these patients are diagnosed with early glaucoma and immediately started on medical therapy, while others are referred to another center for further examination. In either case, a young person is diagnosed or suspected of having a disease that is potentially blinding and requires lifelong treatment and follow-up. This places a serious psychological burden on the patient and their family. In order to avoid misleading patients, ophthalmologists should know and distinguish the effects of anatomical differences on OCT reports.

### Split RNFL and Shifted RNFL Peaks

**Figure 5:**
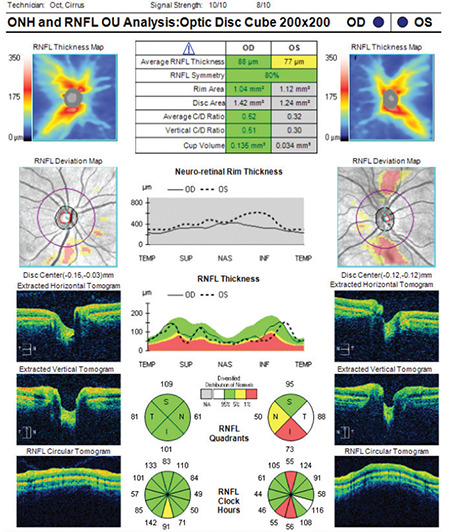
(Cirrus HD-OCT) The superior RNFL bundles of both eyes of this patient show split RNFL defect. On the TSNIT profile, the superior vertex in both eyes is split into two peaks separated by a valley. In addition, a displaced RNFL configuration which is more prominent in the left eye is observed in the inferior quadrants of both eyes. Average RNFL thickness is within normal limits on the right and borderline on the left RNFL: Retinal nerve fiber layer, OCT: Optical coherence tomography

In most individuals, the retinal ganglion cell projections converge towards the superior and inferior poles of the optic disc to form thick RNFL bundles. This anatomical feature is displayed as two peaks on the patient’s expected curve on TSNIT graphs formed according to the normative database. In some cases, the superior and/or inferior RNFL bundles are divided in two and enter the optic disc in the form of a pair of separate bundles each. This is called a split RNFL.^16^ This split RNFL structure was later demonstrated in a histopathological study to be a variation of normal rather than an artifact.^17^ With the widespread use of OCT scans, these types of images have become more common (Figure 5). This variation, which is mostly encountered in young, healthy people, leads to red disease by giving the appearance of a local RNFL defect.

**Figure 6:**
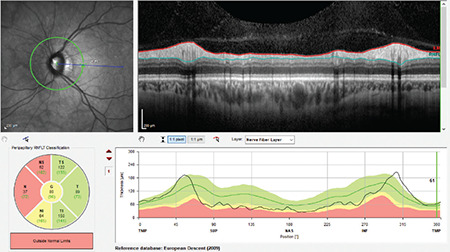
(Spectralis OCT) In the TSNIT profile of a myopic patient, RNFL thickness values in the nasal sectors appear low due to temporal displacement of the RNFL peaks. This occurs because the peaks do not align with expected positions in TSNIT graphs based on the normative database RNFL: Retinal nerve fiber layer, OCT: Optical coherence tomography

Shifted RNFL peaks occur when average RNFL thickness values are within normal limits but the peaks are not aligned with expected positions on TSNIT graphs based on the normative database (Figure 6). In other words, the RNFL bundles have completely normal thickness but abnormal topographic position. Hong et al.^18^ stated that RNFL peaks can be displaced temporally and cause red disease artifact in healthy individuals. Hood et al.^19^ stated that the position of RNFL peaks on TSNIT graphs are in the same region as major retinal vessels. A shifted RNFL can sometimes be caused by cyclotorsion of the eye as well. To compensate for this, the Spectralis OCT has the FoBMO axis (axis between the centers of the fovea and Bruch’s membrane opening) connecting the foveal center and disc center.^20^ The starting and ending points of the TSNIT graph are calculated according to the FoBMO axis.

It is very important that the completely normal split RNFL and shifted RNFL configurations are well recognized to be able to accurately diagnose localized RNFL loss. Careful examination of the RNFL TSNIT profile and checking that optic nerve head parameters and macular scan results are within normal limits is very important for recognizing these anomalies. Split RNFL and shifted RNFL can each exist in both the superior and inferior regions or appear in only one of these quadrants, and the two variations can also be seen together in the same eye. With new software it will be easier to recognize these artifacts in the future.

### Refractive Media Opacities

**Figure 7:**
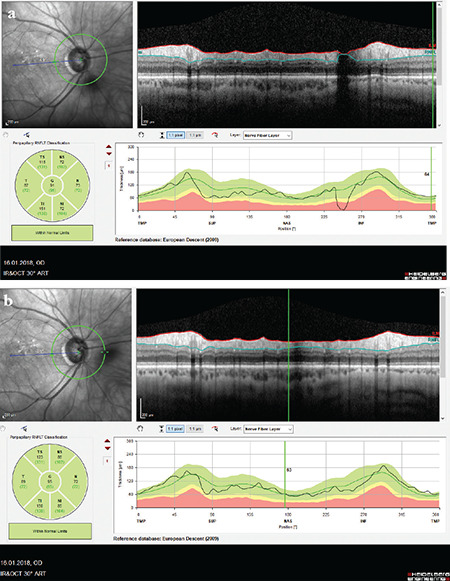
(Spectralis OCT) A Weiss ring coinciding with the laser scanning region causes a segmentation artifact in the inferonasal sector of the right optic disc due to shadowing. On the TSNIT profile, it is seen that RNFL thickness has a value of 0 in this region (a). Upon movement of the eye, the Weiss ring also moved and the device’s algorithm performed segmentation correctly in the inferonasal region. The Weiss ring in the nasal region produces minimal shadowing that does not impair segmentation (b) RNFL: Retinal nerve fiber layer, OCT: Optical coherence tomography

The presence of vitreous opacities such as Weiss ring in the scanning area can cause imaging artifacts, often leading to red disease and sometimes to green disease. Vitreous opacities can also cause the device to incorrectly detect the disc center, resulting in scanning of the wrong area. Because these opacities change position with eye movements, repeated scans may be affected intermittently while appearing normal at other times (Figures 7a and 7b). These artifacts can be recognized by carefully examining the deviation map, IR image, and TSNIT graph. Results may return to normal in scans performed immediately after asking the patient to move their eye from side to side. In addition, cataracts, asteroid hyalosis, and vitreous hemorrhages can also cause refractive media opacities. Refractive media opacities are the most common cause of artifacts in elderly patients.

### Vitreoretinal Interface Problems

### Peripapillary Vitreoretinal Traction and Hyaloid Thickening:

**Figure 8:**
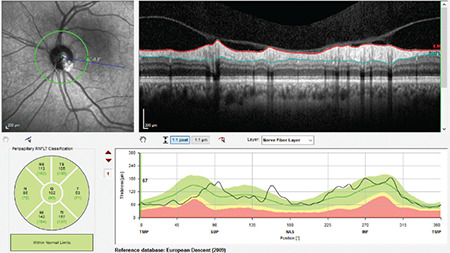
(Spectralis OCT) Areas of pronounced vitreoretinal traction are seen upon examination of the vitreoretinal interface in the RNFL profile of a patient being followed due to ocular hypertension. In the TSNIT profile, RNFL thickness is higher than expected normal values in certain areas in the superonasal region. Peripapillary RNFL thickness is classified as above normal in most sectors RNFL: Retinal nerve fiber layer, OCT: Optical coherence tomography

**Figure 9:**
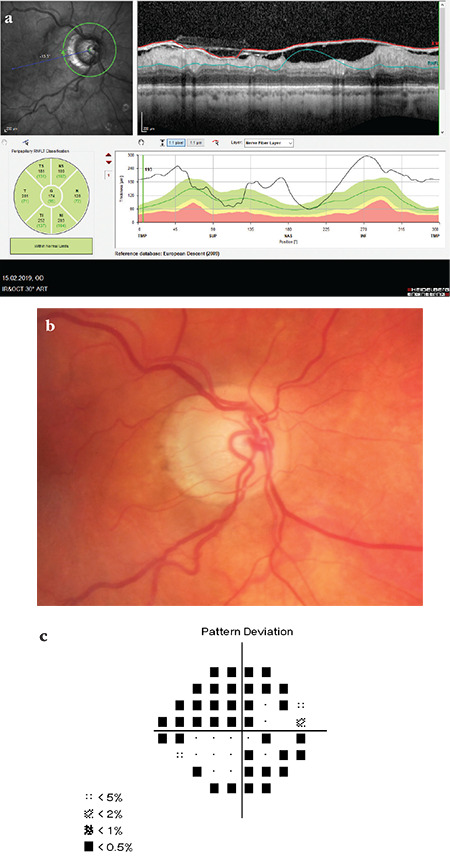
(Spectralis OCT) Segmentation is completely disrupted in a patient with substantial ILM thickening. Peripapillary RNFL classification appears to be within normal limits in all sectors, and analysis of the sectors shows that average RNFL thickness values are much higher than expected average values. The same findings are seen in the TSNIT profile (a). Disc photography shows prominent glaucomatous pitting and peripapillary atrophy (b). There is significant glaucomatous visual field loss in the same eye (c). Note that OCT RNFL classification appears to be within normal limits in all sectors RNFL: Retinal nerve fiber layer, OCT: Optical coherence tomography

Vitreoretinal traction can cause pseudothickening of the RNFL, resulting in green disease artifact (Figure 8). This situation can arise when posterior vitreous detachment is developing in healthy eyes, or RNFL thickness measurement may be artificially high due to posterior hyaloid thickening in conditions such as advanced diabetic retinopathy. Segmentation errors may also occur in such cases (Figure 9a). On sector analysis, average RNFL thickness values may be much higher than expected. Marked glaucomatous changes may be observed on optic disc analysis (Figure 9b). Visual field testing may also reveal glaucomatous visual field loss (Figure 9c). When the vitreous completely detaches from the retina, RNFL thickness decreases significantly and the actual values become apparent.

### Optic Nerve Head Drusen

**Figure 10:**
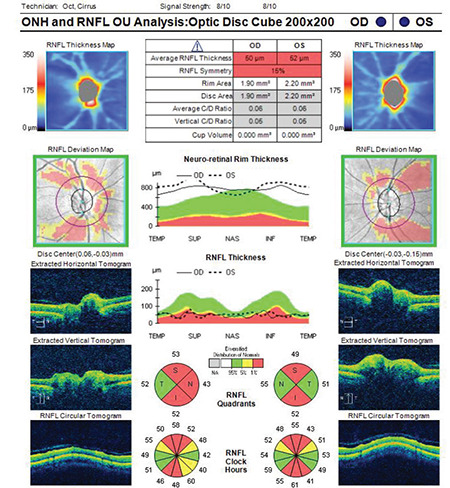
(Cirrus HD-OCT) Quality score is within normal limits in a scan from a patient with optic nerve head drusen. However, note that although the diameter of the disc is normal, the cup volume value is 0. Despite significant RNFL thinning, neuroretinal rim thickness is higher than normal values in optic nerve head measurements RNFL: Retinal nerve fiber layer, OCT: Optical coherence tomography

In patients with optic nerve head drusen, scan quality score is within normal limits and no artifacts are seen (Figure 10). An important clue is that cup area or volume is very small or at a value of 0 despite normal disc size. In optic nerve head measurements, neuroretinal rim thickness above normal values is conspicuous. In addition to RNFL measurements, macular scans and visual field testing for the detection of glaucomatous damage assist diagnosis. In such cases, progression analysis can be used if there is suspicion or diagnosis of glaucoma, but it should be noted here that optic disc drusen themselves may also cause progressive RNFL and visual field losses similar to glaucoma.^21,22^ It is also beneficial to use other tests for the diagnosis of disc drusen.

### Large Disc

In case of oversized optic nerve head, the peripapillary RNFL scanning ring will pass close to the disc margin, leading to inaccurate results. It should not be forgotten that these discs may present with other anomalies such as tilted disc and peripapillary atrophy, and macular scans and visual field testing should be preferred as much as possible during follow-up.

### Chorioretinal Scarring

Chorioretinal scars, especially those located close to the disc, cause localized RNFL losses depending on the size of the lesion. These losses are mostly sectoral and also manifest on visual field as localized absolute scotomas. It should be remembered that macular scans may also be affected, and fundus scanning should be performed carefully to rule out these lesions.

### Other Artifacts That Cause Green Disease

In addition to the aforementioned diseases, green disease may also appear in the form of thinning in certain sectors in eyes with substantially high RNFL thickness values. Because the patient has very high RNFL thickness values initially, regions with glaucomatous damage will be classified as green according to the normative database. In such cases, the importance of progression analysis becomes clear once more. This allows patients to be identified as progressive while their results are green.

In uveitic patients, edema causes RNFL thickening and measured values may be high. This in turn may mask glaucomatous RNFL thinning.^23^

**Figure 11:**
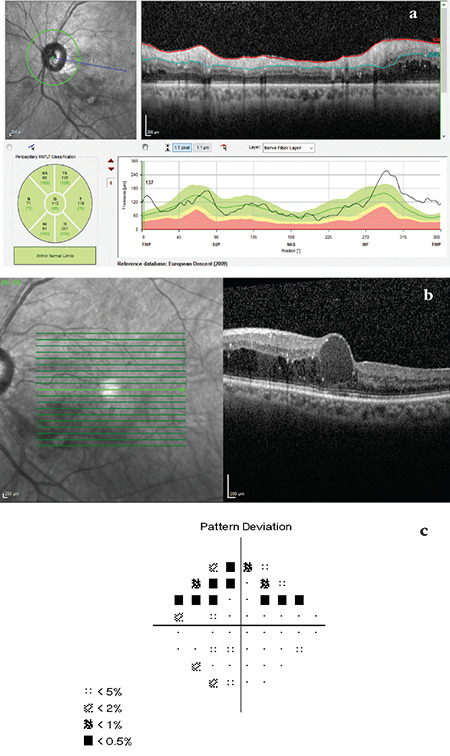
(Spectralis OCT) All sectors are green in the RNFL classification graph of a glaucoma patient with diabetic retinopathy and macular edema. In the TSNIT profile, the thickness curve is seen to be above normal limits in the inferotemporal, temporal, and superotemporal regions (a). Macular OCT analysis shows diabetic macular edema (b). Central 24-2 visual field testing demonstrates glaucomatous superior arcuate defect (c). Note that the sector classifications are completely within normal limits in the OCT RNFL examination of this patient RNFL: Retinal nerve fiber layer, OCT: Optical coherence tomography

In diabetic macular edema, RNFL thickness measurements may be high due to the retinal edema despite the presence of glaucomatous damage (Figure 11a), and green classification in these sectors may obscure the glaucomatous damage. Macular scanning with OCT facilitates the detection of diabetic macular edema (Figure 11b). In such cases, when the information provided by structural tests is limited, visual field testing may allow us to detect glaucomatous damage (Figure 11c).

In age-related macular degeneration, retinal edema can cause green disease as in uveitic and diabetic cases.

Epiretinal membranes can also cause artificially high RNFL thickness measurements and result in green disease.

**Figure 12:**
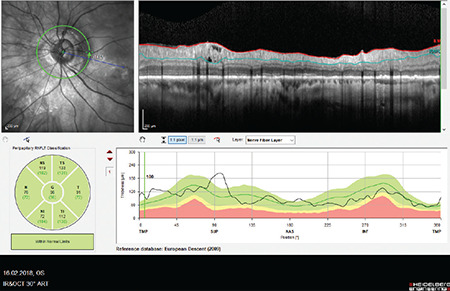
(Spectralis OCT) Peripapillary retinoschisis in the superior quadrant is apparent in a myopic patient (see RNFL profile image in the upper right). Note that the RNFL thickness curve is well above expected values in the location corresponding to the region of retinoschisis in the TSNIT profile RNFL: Retinal nerve fiber layer, OCT: Optical coherence tomography

In peripapillary retinoschisis, there is a temporary increase in RNFL thickness (Figure 12).^24,25^ Values return to normal after the resolution of retinoschisis. It should be noted that the coexistence of peripapillary retinoschisis and glaucoma is common.

The low postoperative IOP values of patients who have undergone surgery may also cause RNFL thickness to appear increased. This should be taken into account when performing progression analysis.

### How to Avoid Overlooking Artifacts

### The Whole Report Should Be Evaluated, Including Raw Data if Necessary

Most of the time, interpretations are made without looking at the entire report. In the course of fast-paced practice, the physician usually makes interpretations by looking at colored maps and graphs. It is only possible to catch the aforementioned artifacts when one looks at the entire report and, in suspicious cases, at the raw data. When an artifact is detected, it must be decided whether it is a localized artifact or one that affects the test in general, and the test should be repeated if necessary. As with artifacts associated with cataract, some artifacts cannot be avoided by repeating the test.

### Output Data Should Be Consistent with Clinical Presentation

Sometimes the patient’s clinical signs say it all. For example, although we may know very well that the patient has advanced glaucomatous damage, all quadrants and sectors may be green in the OCT report, or vice versa. By examining the report in detail and the raw data in the device, it can be understood whether these OCT findings are due to an artifact. 

### Technicians Should Be Trained to Distinguish Major Artifacts

A well-trained technician recognizes most artifacts that occur during a scan, whether they stem from device settings or are patient-based, and repeats the test after correcting the underlying cause. This prevents wasting of time and effort.

### Ocular Comorbidities Should Be Considered

It should be kept in mind that ocular disorders such as diabetic macular edema, uveitic cystoid macular edema, epiretinal membrane, age-related macular degeneration, and macular edema due to postoperative hypotonia can directly affect OCT results.

### Diagnosis Should Not Be Based on a Single Test Result or Region Scan

One must bear in mind that artifacts may be present in every test; therefore, important decisions regarding treatment and follow-up should not be based on a single report or a single region scan. It should also be ensured that optic disc, RNFL, and macular analyses are consistent with one another.
